# Economically Optimal Rate for Nutrient Application to Maize in the Semi-deciduous Forest Zone of Ghana

**DOI:** 10.1007/s42729-020-00240-y

**Published:** 2020-04-17

**Authors:** Benedicta Essel, Robert Clement Abaidoo, Andrews Opoku, Nana Ewusi-Mensah

**Affiliations:** 1 Department of Crop and Soil Sciences, Faculty of Agriculture, Kwame Nkrumah University of Science and Technology (KNUST), Kumasi, Ghana; 2 CSIR - Soil Research Institute, Academy Post Office, Kwadaso, Kumasi, Ghana; 3 Department of Theoretical and Applied Biology, Kwame Nkrumah University of Science and Technology (KNUST), Kumasi, Ghana; 4 International Institute of Tropical Agriculture, PMB 5320, Oyo Road, Ibadan, Nigeria

**Keywords:** Fertilizer response, Optimization, Asymptotic function, Net return to fertilizer, Nutrient use efficiency, Cost to grain price ratio

## Abstract

Low inherent nitrogen (N), phosphorus (P), and potassium (K) contents of smallholder farms limit maize grain yield. Maize grain yield response to N, P, and K mineral fertilizer application and economically optimal rates for nitrogen (EOR^N^), phosphorus (EOR^P^), and potassium (EOR^K^) were evaluated on a Ferric Acrisol within the semi-deciduous forest zone of Ghana. The nutrient rates evaluated were N (0, 30, 60, 90, and 120 kg N ha^−1^), P (0, 30, 60, and 90 kg ha^−1^ P_2_O_5_), and K (0, 30, 60 and 90 kg ha^−1^ K_2_O). The treatments were arranged in a randomized complete block with three replications using an incomplete factorial design. Nutrient responses were determined using asymptotic quadratic-plus plateau functions. The best nitrogen rate for all P and K levels was 60 kg ha^−1^, which gave grain yield of 5 t ha^−**1**^**.** Nitrogen uptake, N agronomic and N recovery efficiencies peaked at 60 kg N ha^−**1**^ while N partial factor productivity declined with increasing N application rate. Cost to grain price ratios (CP) were 1.29, 1.65, and 1.65 for N, P, and K, respectively. The EOR^N^ was 61 kg ha^−1^, 32% less than the recommended 90 kg N ha^−1^ for maize production in the semi-deciduous forest zone of Ghana. Nitrogen application had the lowest CP ratio, making its application economically profitable than P and K. The findings suggest that the application of N at 61 kg N ha^−1^ to maize is economically profitable than at higher application rates. However, further studies should be conducted on farmers’ fields to validate the results obtained.

**Electronic supplementary material:**

The online version of this article (10.1007/s42729-020-00240-y) contains supplementary material, which is available to authorized users.

## Introduction

Maize is the most important cereal crop in Ghana with an estimated annual production of 750,000 ha (Tetteh et al. 2017). The crop is predominantly cultivated by smallholder resource-constrained farmers, mostly under rain-fed conditions. Despite the large hectares of farmland cultivated yearly, the average grain yield of maize on farmers’ fields in Ghana in 2014 was 1.7 t ha^−1^ (FAOSTAT 2019) which is approximately 253% less than the estimated achievable yield of about 6 t ha^−1^ reported by Adu et al. (2014). These low yields could be due to several factors including low soil fertility, drought during critical stages of crop growth, weed and pest infestation, and limited use of inputs such as fertilizer and improved seeds (Adu et al. 2014).

Sanchez (2002) reported that soil fertility depletion in smallholder farmers’ fields was the major biophysical cause for the declining rate of crop production in most sub-Saharan African countries. Intensive agriculture cannot be sustained unless nutrients are applied to the soil to replace the ones removed through crop production (Morris et al. 2007). The most common sources of nutrients to crops are mineral and organic fertilizers.

Fertilizer use in Ghana is low and the recommended rates for application are usually blanket (Tetteh et al. 2017). Moreover, climate change has altered the soil biophysical environment and impoverished the soils to the extent that the efficiency of blanket fertilizer application has become questionable (Okebalama 2014). The fertilizer recommendation for maize in Ghana was last updated in 1974; since then, efforts have been made to update these recommendations (Tetteh et al. 2017). However, no absolute conclusions have been made yet. The fertilizer recommendation for maize was 64:38:38 kg ha^−1^ N:P_2_O_5_:K_2_O (Agricultural Extension Handbook 1977). Safo (1990) reported 67:45:45 kg ha^−1^ N:P_2_O_5_:K_2_O as the rate recommended by the Ministry of Food and Agriculture, Ghana. These fertilizer recommendations were updated to 90:60:60 kg ha^−1^ N:P_2_O_5_:K_2_O by Tetteh et al. (2008). In spite of all these recommendations, fertilizer application in Ghana was approximately 8 kg ha^−1^ (FAO 2005). This was further increased to 34 kg ha^−1^ in 2012, which was still far below the 50 kg ha^−1^ recommended by the Abuja Summit (Fening 2015). It was observed that smallholder farmers who largely depend on low external inputs, hardly adhere to the recommended fertilizer application rates (Tetteh et al. 2017). This is largely due to the high cost involved, which is usually beyond the purchasing power of most farmers. Even at the current subsidized rates, fertilizers are still expensive to the smallholder farmer (Tetteh et al. 2017).

As a way to counter the use of blanket recommendations, Optimizing Fertilizer Recommendations for Africa (OFRA) was introduced in 2012. Chapoto and Tetteh (2014) reported that yield margins obtained from applying optimum fertilizer rates to maize crops were 148–248% greater than using blanket recommendation. This is because most of the recommended rates of fertilizer application have been found to be generally high as compared to the economically optimal rates (EOR) of nutrients determined from the results of field research conducted across the 13 sub-Saharan African countries.

In many developing countries, there is still a large gap between the economically achievable yield and the average yield (Roy et al. 2006). This is mostly due to the fact that fertilizer recommendations are not adequately profit-oriented and do not consider the financial status of the smallholder farmer (Cyamweshi et al. 2018). Optimizing fertilizer use to obtain the optimum economic yield reduces nutrient loss to the environment (Webb 2010) and is therefore an important strategy to improve nutrient use efficiency in crops.

Adoption of fertilizer optimization is at the early stages in most sub-Saharan Africa countries with Kenya, Tanzania, and Uganda having considerable experience in this domain (Rware et al. 2017). This current study in Ghana has become necessary because despite the substantial research conducted by Tetteh et al. (2017) in the Forest/Savanna Transitional agro-ecological zone (AEZ), Guinea Savannah and Sudan Savannah AEZs, information on fertilizer optimization in the semi-deciduous forest AEZ of Ghana is lacking.

Studies aimed at predicting the economically optimal rates of nutrient application to crops led to several reports hypothesizing that curvilinear response functions (Jansen et al. 2013), quadratic-plus-plateau models (Cerrato and Blackmer 1990), asymptotic curvilinear-plateau models (Wortmann and Sones 2017) best described crop yield response to fertilizer application. This study however hypothesizes that the OFRA fertilizer optimization model uses a nonlinear regression function (asymptotic quadratic-plus-plateau model) to predict EOR of nutrients for crops. The objectives of the research were to (i) quantify the grain yield response of maize to N, P, and K application; (ii) evaluate N use efficiency of maize; (iii) determine the net returns to fertilizer use; and (iv) determine the economically optimal nutrient rates for N (EOR^N^).

## Materials and Methods

### Description of the Study Site

The study was conducted under rain fed conditions in the major and minor rainy seasons of 2017 at the plantation section of the Department of Crop and Soil Sciences, Faculty of Agriculture, Kwame Nkrumah University of Science and Technology (KNUST), Kumasi, Ghana. Fertilizer treatments were applied in the major season, whereas the minor season experiment was conducted to evaluate the residual effect of the fertilizer application. The site is located on latitude 6° 40′ 59.364″ N and longitude 1° 33′ 3.618″ W at an elevation of 287 m asl in the semi-deciduous forest AEZ of Ghana. Soils of the study site have been classified as Ferric Acrisols and belongs to the Kumasi series, developed over a deeply weathered granite (FAO/UNESCO 1998). The average annual rainfall of the study site is 1500 mm.

### Experimental Design and Treatments

The nutrients evaluated in the study were N (0, 30, 60, 90, and 120 kg ha^−1^ N), P (0, 30, 60, and 90 P_2_O_5_ kg ha^−1^) and K (0, 30, 60 and 90 K_2_O kg ha^−1^). The experimental design used was incomplete factorial arranged in randomized complete block with three replications. The incomplete factorial was chosen to reduce the number of plots considering the number of treatments. A total of twelve treatments (N:P_2_O_5_:K_2_O (kg ha^−1^) of T_1_–0:0:0, T_2_–0:60:60, T_3_–30:60:60, T_4_–60:60:60, T_5_–90:60:60, T_6_–120:60:60, T_7_–90:0:60, T_8_–90:30:60, T_9_–90:90:60, T_10_–90:60:0, T_11_–90:60:30, and T_12_–90:60:90) were used in the major and minor seasons trials. The plot sizes were 3 × 4 m. Maize response to N application was determined using treatments which received the recommended rates of P and K (T_2_, T_3_, T_4_, T_5_, and T_6_). Phosphorus response was determined using T_5_, T_7_, T_8_, and T_9_ and K response using T_5_, T_10_, T_11_, and T_12_. Maize variety *Omankwa*, an early-maturing (90–95 days), drought tolerant, quality protein maize with a yield potential of 5 t ha^−1^ was used as the test crop. This is the most preferred variety by farmers in the study area.

### Soil Sampling and Laboratory Analysis

Soil samples were collected with a cylindrical core sampler at a depth of 0–15 cm before sowing and at physiological maturity (R_6_). Before sowing, 10 soil cores were sampled at the experimental site, bulked and sieved to pass through a 2-mm mesh sieve for laboratory analyses. Undisturbed soil samples were used for bulk density determination. The physical and chemical analyses were carried out following standard procedures as described by Motsara and Roy (2008).

### Crop Management

The experimental site was left fallow and dominated with *Panicum maximum* before the onset of this experiment. The experimental site was disc plowed and followed by secondary tillage of disc harrowing at an approximate depth of 15–20 cm. Maize seeds were sown on April 10, 2017, and September 13, 2017 for both the major and minor season experiments, respectively, at a spacing of 75 × 40 cm at two seeds/stand to attain a plant population density of 66,666 plants/ha.

The fertilizers applied were urea, triple superphosphate and muriate of potash to supply N, P, and K, respectively. All three fertilizers were applied manually through band placement at 2 weeks after sowing (WAS). Urea was, however, applied in two splits with 75% applied at 2 WAS and the remaining 25%, at 5 WAS. Weed management was done by hand hoeing twice within the season. Pest management was done within the cropping cycle using Bypel 1 (PrGV.Bt) at 15 g/ 15 L of water to control the fall army worm: *Spodoptera frugiperda* (J. E. Smith) (Insecta: Lepidoptera: Noctuidae).

At physiological maturity, maize plants within the three central rows of each plot in a net plot area of 4.5 m^2^ were harvested for grain yield determination. Maize grains were adjusted to 15% moisture content (Lauer 2002).

Nitrogen uptake was computed under varying rates of P and K. The other nutrient use efficiency indices such as N agronomic efficiency (NAE), N partial factor productivity (NPFP), and N recovery efficiency (NRE) were computed under P and K rates of 60 kg P_2_O_5_ ha^−1^ and 60 kg K_2_O ha^−1^. Nitrogen uptake was determined from the equation

1 $$ \left(\mathrm{N}\ \mathrm{uptake}=\mathrm{grain}\ \mathrm{N}\ \left(\%\right)\times \mathrm{grain}\ \mathrm{yield}\right) $$

Afterwards, NAE, NPFP, and NRE were also determined using the following formulae by Dobermann (2005):

2 $$ \mathrm{NAE}=\left({\mathrm{Y}}_{+\mathrm{N}}-{\mathrm{Y}}_{\mathrm{N}0}\right)/\mathrm{Nrate} $$

3 $$ \mathrm{NPFP}=\mathrm{Y}/\mathrm{Nrate} $$

4 $$ \mathrm{NRE}=\left({\mathrm{UN}}_{+\mathrm{N}}-{\mathrm{UN}}_{\mathrm{N}0}\right)/\mathrm{Nrate} $$

where Y_+N_ = grain yield of the fertilized plot; Y_N0_ = grain yield of the unfertilized plot (N_0_); Y = grain yield; UN_+N_ = nitrogen uptake of the fertilized plot, and UN_N0_ = nitrogen uptake of the unfertilized plot (N_0_).

### Statistical Analysis

The data obtained from the study was subjected to analysis of variance using Statistix 10 (Analytical Software, Tallahassee, FL, USA) (Statistix 2013). Post hoc comparison of treatment means was then carried out using the least significant difference method at *p* value of 0.05. Among the several models (exponential, linear-plus-plateau, quadratic, quadratic-plus-plateau, and square root) commonly used to describe maize response to N fertilizer application, Cerrato and Blackmer (1990) reported that the quadratic-plus-plateau model best described maize yield response to N application by predicting lower economically optimal rates of nutrient application as compared to the other models. In view of this, asymptotic quadratic-plus-plateau yield functions were determined in relation to nutrient rate effects, using nonlinear regression model as follows:

$$ \mathrm{Yield}\ \left(\mathrm{Mg}/\mathrm{ha}\right)=\mathrm{a}\hbox{-} {\mathrm{bc}}^{\mathrm{N}}, $$

where a = yield at the plateau or maximum yield, b = gain in yield due to nutrient application, c^N^ = determines the shape of the quadratic response where c = curvature coefficient and N = nutrient rate.

Economically optimal nutrient rates (EORs) were determined using the OFRA fertilizer optimization tool developed by University of Nebraska, Lincoln, USA. Net returns to fertilizer use were also determined using the OFRA fertilizer optimization tool. These were dependent on the grain yield, grain value (US$0.43 kg^−1^) and fertilizer value (US$0.55 kg^−1^) in 2017. Nonlinear regression analysis was also used to derive an equation to relate EOR^N^ to a varying range of fertilizer use cost to grain price ratios (CP), using a grain value of US$0.43 kg^−1^. The EOR^N^ was determined at CP ratios of 1.00, 1.50, 2.00, and 2.50, with CP as the independent variable.

## Results

### Characterization of Soils at the Experimental Site

The initial physicochemical properties of the soil at the experimental site are presented in Table 1. The results indicate that the soil was loamy sand in texture with a low bulk density (1.21 Mg m^−3^), gravimetric moisture content of 9.18% and porosity of 42.89%. The soil was moderately acidic (pH = 5.90) with very low organic carbon (1.12%), very low total N (0.09%), and a low level of available Bray 1 phosphorus concentration (15.31 mg kg^−1^ soil). The initial exchangeable calcium, magnesium, potassium, and sodium levels were low (4.81 cmol_(+)_ kg^−1^ soil), moderate (2.41 cmol_(+)_ kg^−1^ soil), low (0.31 cmol_(+)_ kg^−1^ soil), and low (0.17 cmol_(+)_ kg^−1^ soil), respectively. The exchangeable acidity was low (0.40 cmol_(+)_ kg^−1^ soil), and the effective cation exchange capacity was also low (7.43 cmol_(+)_ kg^−1^ soil).

**Table 1:** Initial physicochemical properties of the soil (0–15 cm) at the experimental site before sowing

Soil property	Value
Physical properties	
Sand (%)	80.00
Silt (%)	14.00
Clay (%)	6.00
Texture	Loamy sand
Bulk density (Mg m^−3^)	1.21
Soil moisture content (%)	9.18
Soil porosity (%)	42.89
Chemical properties	
Soil pH (1:2.5, H_2_O)	5.90
Soil organic carbon (%)	1.12
Total N (%)	0.09
Available Bray 1 P (mg kg^−1^)	15.31
Exchangeable bases (cmol_(+)_ kg^−1^)	
Ca^2+^	4.81
Mg^2+^	2.14
K^+^	0.31
Na^+^	0.17
Exchangeable acidity (Al^3+^ + H^+^) (cmol_(+)_ kg^−1^)	0.40
ECEC (cmol_(+)_ kg^−1^)	7.43
Base saturation (cmol_(+)_ kg^−1^)	94.89

*ECEC* effective cation exchange capacity

Generally, the overall fertility status at the experimental site was poor with most of the measured soil properties being lower than the critical values of nutrients required for crop growth, according to the ratings by Landon (2014).

### Maize Grain Yield Response to Mineral Fertilizer Application

The effect of nitrogen, phosphorus, and potassium fertilizer application rates on the observed grain yield of maize during the major season and minor season of 2017 are presented in Table 2. The results indicate that the application of N and P fertilizers significantly (*p* ≤ 0.05) increased observed grain yields of maize in the major season of 2017. The application of K, however, did not have any significant (*p* = 0.98) effect on observed grain yield. The average grain yield for N_0_, P_0_ and K_0_ were 3.68, 4.44, and 4.37 t ha^−1^, respectively. The lowest grain yield was observed in N_0_ (3.68 t ha^−1^) while the highest was observed under N_60_ (5 t ha^−1^) which was at par with that of P_60_ (5 t ha^−1^).

**Table 2:** Maize grain yield (Mean ± SE) response to all N, P, and K application levels in the 2017 major and minor cropping seasons

Season/application rates	0	30	60	90	120
2017 major	Nitrogen (kg ha^−1^ N)^†^				
	3.68 ± 0.43 b	4.32 ± 0.60 ab	5.00 ± 0.60 a	4.67 ± 0.24 a	4.17 ± 0.60 ab
	Phosphorus (kg ha^−1^ P_2_O_5_)^‡^				
	4.44 ± 0.45 ab	3.93 ± 0.61 b	5.00 ± 0.25 a	4.10 ± 0.61 ab	–
	Potassium (kg ha^−1^ K_2_O)^§^				
	4.37 ± 0.45a	4.34 ± 0.61a	4.27 ± 0.25 a	4.49 ± 0.61 a	–
CV (%)	16.46				
2017 minor	Nitrogen (kg ha^−1^ N)^†^				
	2.66 ± 0.32 a	2.03 ± 0.45a	2.72 ± 0.45 a	2.61 ± 0.18 a	2.64 ± 0.46 a
	Phosphorus (kg ha^−1^ P_2_O_5_)^‡^				
	2.37 ± 0.33 a	2.12 ± 0.46 a	2.77 ± 0.18 a	2.87 ± 0.46 a	–
	Potassium (kg ha^−1^ K_2_O)^§^				
	2.35 ± 0.33 a	2.53 ± 0.46 a	2.48 ± 0.18 a	2.77 ± 0.46 a	–
CV (%)	21.21				

Values are means of three replicates. Means with the same letters in a row are not significantly different from each other according to Least significant difference method of mean separation

*CV* coefficient of variation, *SE* standard error

^†^Effect of N at all levels of P and K

^‡^Effect of P at all levels of N and K

^§^Effect of K at all levels of N and P

**Fig. 1:**
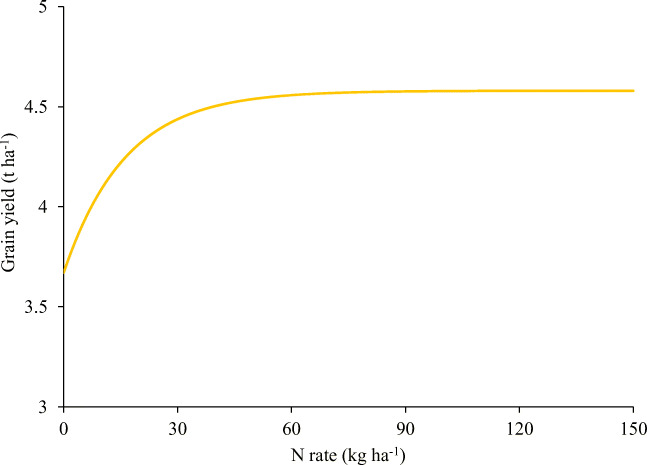
Response of maize to fertilizer N application (2017 major season)

Unlike the 2017 major season where the application of N and P fertilizers significantly (*p* ≤ 0.05) increased observed grain yields of maize during the cropping period, the observed grain yields obtained in the 2017 minor season were not significantly different (*p* > 0.05) from each other. The grain yields obtained in the 2017 minor season ranged between 2.03 and 2.87 t ha^−1^ (Table 2). Among the three nutrient elements (N, P, and K) applied, only the response of grain yield to applied N in the 2017 major season followed a typical curvilinear to plateau nature (Fig. 1). There was a steep increase in maize grain yield response to N application, followed by lower rates of expected yield increases at higher rates of N application until a plateau was reached followed by a subsequent yield decline (Table 3). The grain yield of maize increased with increasing N rate until a plateau was reached at 60 kg ha^−1^ N application after which the grain yield stabilized (Fig. 1). The increases in observed grain yield with N application ranged from 0.49 to 1.32 t ha^−1^, corresponding to relative increment of 13–36% over the control in the 2017 major season. The resulting yield response function with respect to N application was as follows:

5 $$ \mathrm{Yield}=4.58-0.91\left({0.94}^{\mathrm{N}}\right) $$

**Table 3:** Asymptotic response functions and recommended N rate for maize in the semi-deciduous forest zone of Ghana

Grain yield (t ha^−1^)							Recommended N rate	
Response coefficients			N rate of change (kg ha^−1^)					
*a*	*b*	*c*	0–30	30–60	60–90	90–120	EOR^†^	CFR^‡^
t ha^−1^							kg ha^−1^	
			Expected yield increases (t ha^−1^)					
4.58	0.91	0.94	0.77	0.12	0.02	0	61	90
*R*^2^ = 0.62								

*a* yield at the plateau or maximum yield, *b* gain in yield due to nutrient application, *c*^*N*^ determines the shape of the quadratic response where *c* is the curvature coefficient and *N* is the nutrient rate

CFR: current fertilizer recommendation for maize used was 90:60:60 N:P_2_O_5_:K_2_O kg ha^−1^. EOR: economically optimal rate was determined using 50 kg urea at GH¢110, TSP at GH¢150, and MOP at GH¢150, equivalent to approximately, US$27.50, US$37.50, and US$37.50, respectively. Maize grain value used was 0.43 kg^−1^

Due to the curvilinear to plateau nature of maize response to fertilizer N application, asymptotic response function was used to predict the maximum grain yield and yield increase of maize in response to N application. Results of the predicted asymptotic quadratic-plus-plateau yield functions in relation to nutrient rate effects are presented in Table 3.

The predicted maximum maize grain yield was 4.58 t ha^−1^ with an average grain yield increase of 0.91 t ha^−1^ resulting from N application. The expected yield increases for N following elemental nutrient rate change from 0 to 30, 30 to 60, 60 to 90, and 90 to 120 kg N ha^−1^ were 0.77, 0.12, 0.02, and 0 t ha^−1^, respectively. The economically optimal rate of N (EOR^N^) was 61 kg N ha^−1^, which is 32% lower than the recommended rate of N required for maize production in the semi-deciduous forest zone of Ghana.

The observed grain yields and those predicted with fertilizer optimization tool, and the net returns from fertilizer use are presented in Table 4. The application of 61 kg N ha^−1^ is recommended since it gave over 2% higher net returns to fertilizer use (US$348.77) compared to 90 kg ha^−1^ recommended N application rate (US$340.31) for maize in the semi-deciduous forest zone of Ghana. The results therefore show superiority of the EOR to the recommended rate of N for maize.

**Table 4:** Grain yield of maize and net returns on investment from N application

Treatment	Grain yield (t ha^−1^)		Net returns to fertilizer use (US$)	Net returns to cost for 1 kg added nutrient (US$)
	Predicted from FOT	Observed from field experiment		
Control (0 kg ha^−1^ N)	3.67	3.68	–	–
EOR (61 kg ha^−1^ N)	4.56	5.00	348.77	0.04
REC (90 kg ha^−1^ N)	4.58	4.67	340.31	−0.83
Yield potential of maize variety used	5.00			

*EOR* economically optimal rate of nitrogen application, *REC* recommended N application rate

### Effect of Fertilizer Application on Nutrient Uptake and Use Efficiency Indices

The observed effects of N application on N uptake and N use efficiency indices of maize grains at the end of 2017 major season are presented in Table 5. It was generally observed that whiles N uptake and NPFP were significantly (*p* ≤ 0.05) influenced by the varying N application rates, there was insufficient evidence to declare significant differences for NAE and NRE.

**Table 5:** Mean effect of N application on N uptake and use efficiency indices in 2017 major season

N rate (kg ha^−1^)	N uptake (kg ha^−1^)	NAE (kg kg^−1^ N ha^−1^)	NPFP (kg kg^−1^ N ha^−1^)	NRE (%)
0	48.55			
30	59.32	14.88	161.56	25.99
60	74.35	18.92	92.26	38.05
90	67.07	4.68	53.58	10.65
120	62.20	2.54	39.21	8.90
Fpr.	0.020	0.62	0.0001	0.07
a	67.27			
b	19.12			
c	0.95			
R^2^	0.72		0.99	
EOR^N^	66.4		91.57	

N uptake was computed under varying rates of P and K. The rest were computed under optimum P and K rates of 60 kg ha^−1^ P_2_O_5_ and 60 kg ha^−1^ K_2_O, respectively. The EOR^N^ (economically optimal N rate) was 61 kg ha^−1^ for a fertilizer N use cost to farm gate price ratio (CP) of 1.3. Asymptotic response coefficients could not be determined for NAE, NPFP, and NRE due to lack of good fit

*NAE* nitrogen agronomic efficiency, *NPFP* nitrogen partial factor productivity, *NRE* nitrogen recovery efficiency, *a* yield at the plateau or maximum yield, *b* gain in yield due to nutrient application, *c* curvature coefficient

Nitrogen uptake in the maize grains increased in response to N_0_ up to N_60_ after which N uptake began to decline. The relative increase in N uptake over the control (N_0_) in response to increasing N rates was within the range of 22.18 to 53.14%, with N_60_ recording the highest increase in uptake over the control. Similarly, NAE increased with every 30 kg increase in N application rate to 60 kg N ha^−1^, after which NAE began to decline. Nitrogen agronomic efficiency in the maize grains increased by 27.08% from 30 to 60 kg N ha^−1^. Nitrogen recovery efficiency also increased by 46.40% when N application rate was increased from 30 to 60 kg N ha^−1^, after which it began to decline. Partial factor productivity decreased with each additional increment in N application rate. The relative decrement in NPFP ranged from − 46.40 to − 65.76%. From the results obtained, the EOR^N^ for N uptake and NPFP were 66.40 kg N ha^−1^ and 91.57 kg N ha^−1^, respectively. Unlike N uptake which was best fitted to an asymptotic regression, NPFP was best fitted to a polynomial regression function due to the continuous decrease in NPFP trend observed. Thus,

6 $$ \mathrm{NPFP}=0.0153{\mathrm{N}}^2-3.6412\mathrm{N}+256.75 $$

### Net Returns to Fertilizer Use

**Fig. 2:**
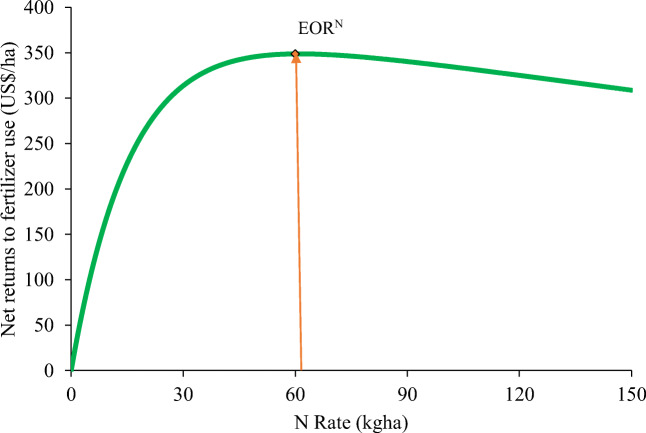
Net returns to nitrogen fertilizer use. This figure is dependent on grain values and fertilizer use costs. Grain value was US$0.43 kg^−1^ for maize. Fertilizer use cost was US$0.55 kg^−1^ for urea (US$1 = GH¢4.00)

The results of net returns to fertilizer use are presented in Fig. 2. Generally, it was evident that the net returns to N fertilizer use increased with an increase in N application rate till a point where further addition of nutrients resulted in a reduction in net returns and consequently leading to financial loss.

### Economically Optimal Nutrient Rates for N over a Varying Range of Cost Price to Grain Ratios

**Fig. 3:**
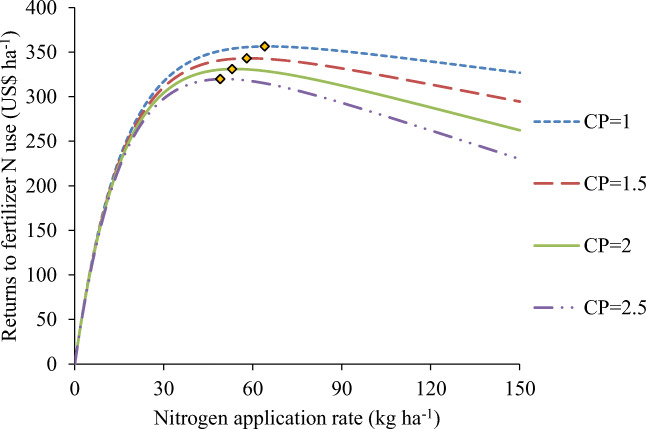
Net returns of maize to fertilizer N application to at varying N rates and fertilizer cost to grain price ratios (CP). The EORN with each CP is indicated by a diamond symbol at the peak of each curve

The average EOR^N^ of maize over varying cost price to grain ratio (CP) ranging from 1.00 to 2.50 are presented in Fig. 3. The average EOR^N^ ranged from 65 to 50 kg N ha^−1^ with CPs of 1.00 to 2.50 (Fig. 3). The EOR^N^ was economically more profitable as the CP decreased. From the results obtained, the EOR^N^ can be estimated from the nonlinear regression model with CP as the independent variable in Eq. 7 as follows:

7 $$ {\mathrm{EOR}}^{\mathrm{N}}=31.75-\left(-49.64\right)\times {(0.67)}^{\mathrm{CP}}\left({R}^2=1.00\right) $$

Considering a US dollar to Ghana cedi exchange rate of US$1 = GH¢4.00, the net returns to N applied at EOR^N^ at CP values of 1.00, 1.50, 2.00, and 2.50 were US$356.34, US$343.08, US$331.01, and US$319.81, respectively. The net returns to N applied decreased as the CPs increased due to high fertilizer costs.

## Discussion

### Maize Grain Yield Response to N, P, and K Application

The observed grain yields of maize were generally lower than the 5 t ha^−1^ attainable yield of the maize variety used (CSIR–Crops Research Institute 2011). Despite the relatively low nutrient contents of the soil at the onset of the field work, average grain yields of maize of 3.68, 4.44, and 4.37 t ha^−1^ were recorded under N_0_, P_0_, and K_0_, respectively. This could be due to the distribution pattern of rainfall during the cropping cycle (Online Resource 1, Online Resource 2). In this present study, the average grain yields of maize of 3.68, 4.44, and 4.37 t ha^−1^ recorded under N_0_, P_0_, and K_0_, respectively, are high since the values are greater than the average grain yields of maize (1.7 t ha^−1^) on farmers’ fields in Ghana. It is likely that higher observed maize yields may be recorded on famers’ field with these treatments under similar rainfall conditions since N, P, and K concentrations on farms are lower than on-station. The different rates of P and K application did not significantly (*p* > 0.05) increase grain yield of maize possibly due to the Liebig’s law of the minimum. According to Santiago et al. (2012), one model of nutrient limitation (Liebig’s law of the minimum) postulates that crop growth is controlled by the nutrient in the lowest supply and only increases with added amount of that nutrient. The initial soil pH of 5.9 is also favorable for maize since the crop is known to thrive in soils within a pH range of 5.5 to 6.5 (Sys et al. 1993). This suggests that because P and K were not the nutrients in the lowest supply, increasing rates of P and K addition to the soil could not result in significant differences in the grain yields. Apart from total soil N which was very low (0.09%) at the experimental site before sowing, the available P and exchangeable K were rated low and moderate leading to small and insignificant yield increases from the application of P and K fertilizers. This confirms the assertion by Landon (2014) that marginal level of soil nutrients will lead to small yield increases when adequate rates of fertilizer nutrients are applied.

As was observed in Table 3, on average, 85% of the grain yield increase occurred following the application of 0 to 30 kg N ha^−1^. The minimal yield increments after higher application of N rates indicates that the application of N at lower rates is more beneficial and profitable to the financially constrained smallholder farmer than higher N rates. Similarly, Kaizzi et al. (2014) reported that on average, about 90% of the grain yield increase in upland rice in Burkina Faso, Ethiopia, Ghana and Nigeria occurred following the application of 0 to 50 kg N ha^−1^. The typical curvilinear to plateau nature of the maize response also indicates that the financially constrained smallholder farmer could apply nutrients at rates that would result in maximum grain yield increase without leading to luxurious consumption.

With regard to the residual trial carried out during the minor season of 2017, no significant (*p* > 0.05) yield differences were observed. According to Grant et al. (2016), N fertilizers are mostly applied to soils to make up for the difference between the N that the crop can access from the soil and the N required for optimum crop yield and quality. Nonetheless, not all the N applied is used by crops during the year of application; residual nutrients are left in the soil in either organic or inorganic forms, or lost from the rhizosphere around the crop by pathways such as volatilization, leaching, immobilization, etc. (Grant et al. 2016, Rahimikhoob et al. 2020). It was observed that the nutrients applied in the previous season did not significantly influence the grain yields of subsequent maize crops (Table 2). Contrary to Grant et al. (2016) who observed an increase in seed yield of canola with increasing rate of N application in the previous season, the maize grain yields observed in the present study were inconsistent in the minor season. The magnitude of increases in grain yields in the residual trial carried out in the minor season were also low, indicating that carry-over of nutrients to subsequent seasons or years after no fertilizer application was relatively low. Similar observations were made by Grant et al. (2016). The residual value of mineral fertilizers mostly caused by adsorption and precipitation reactions, especially P supplying ones are noted for their economic importance in the livelihood of the smallholder resource-constrained farmers due to the reduced need for purchasing fertilizers in subsequent years (Daba and Zewedie 2001). Grain yields of maize observed at the end of the residual trial in the minor season were not significantly (*p* > 0.05) increased by different fertilizer application rates. This indicates the diminishing significance of the residual value of soil nutrients with time as reported by Daba and Zewedie (2001).

The predicted average maximum grain yield of 4.58 t ha^−1^ in response to N application, was approximately 8% lower than the attainable yield (5 t ha^−1^) of the maize variety used. Tetteh et al. (2017) predicted maximum grain yields of maize of 3.79, 3.13, and 3.00 t ha^−1^ in response to N application in the Forest/Savanna Transitional zone, Southern Guinea Savanna, and Sudan Savanna AEZs of Ghana, respectively. This resulted in EOR^N^ of 89, 69, and 69 kg ha^−1^, respectively, as compared to the 90 kg N ha^−1^ recommended for maize production. The low grain yields of maize observed in the savannas as compared to the semi-deciduous forest zone could be due to the relatively lower soil nutrient content and mean annual rainfall of the transition and savanna AEZs.

Table 3 shows that the expected yield increases for N following elemental nutrient rate change from 0 to 30, 30 to 60, 60 to 90, and 90 to 120 kg N ha^−1^ were 0.77, 0.12, 0.02, and 0 t ha^−1^, respectively. The results indicated that yield increase due to elemental nutrient rate change generally reduces with increasing rates of N application. The nutrient rate change from 60 to 90 kg N ha^−1^ resulted in a negligible yield increase of 0.02 t ha^−1^ whereas a further increment in N application from 90 to 120 kg N ha^−1^ did not lead to any yield increase. This is a common occurrence in sub-Saharan Africa as Kibunja et al. (2017) and Senkoro et al. (2017) reported no yield increment in maize after the application of 60 to 120 kg N ha^−1^ in Kenya and Tanzania, respectively.

### Effect of Fertilizer Application on Nutrient Uptake and Use Efficiency Indices

Nutrient use efficiency indices are widely used in crop production systems to measure the capability of a given crop to acquire and utilize nutrients for their biological and grain yields (Fageria et al. 2008). At the end of the 2017 major season, it was observed that NRE and NAE were high at lower rates of nutrient application. The results obtained are similar to that of Kaizzi et al. (2014) who alluded that such occurrence could lead to little residual effect for subsequent cropping. It is therefore not surprising that the residual trial carried out in the 2017 minor season resulted in insignificant yield differences at the different N application rates. The highest NRE of 38.05% recorded in this study, is 15% greater than the estimated global NRE for cereals as reported by Raun and Johnson (1999).

Similarly, NPFP also decreased at higher rates of nutrient application, confirming the findings of Kaizzi et al. (2012) who reported that most components of nutrient use efficiency under a maize-based cropping system were higher at EOR^N^ as compared with higher nutrient application rates.

### Net Returns to Fertilizer Use

Smallholder farmers are mostly financially constrained and therefore require high net returns to justify their use of fertilizer on their crops (Wortmann and Sones 2017). From the results obtained, the financially constrained farmer can take advantage of the higher returns accrued from using low nutrient application rates. As can be observed from Fig. 2, the steeper the slope of the response curve, the higher the net returns to fertilizer use. Therefore, as the amount of money invested in purchasing fertilizer increases, the slope decreases until it reaches a plateau and finally declines leading to profit reduction. Generally, the net returns to N fertilizer use decreased as the N fertilizer application rate exceeded the EOR^N^. The results obtained from this study are similar to a report by Maro et al. (2014) in Northern Tanzania who established that net return and value cost ratio decrease as nutrient application rates get further away from the optimum.

Several other authors in sub-Saharan Africa have reported higher net returns to fertilizer use. In Western Kenya, Kibunja et al. (2017), after applying N fertilizer of US$117.94 to maize, accrued net returns of US$343.98 which was profitable (B/C ratio = 1.92). Gittinger (1982) reported that a benefit/cost (B/C) ratio greater than 1 is profitable because the benefits exceed the cost of investment. Net return is dependent on the value of the nutrient applied and hence may vary from place to place. In Burkina Faso, Ouattara et al. (2017) accrued a net benefit of US$ 222.09 after investing US$63.45 in fertilizer use (B/C = 2.5). High net returns accounting for high B/C ratios of 2.25, 2.64, and 2.82 were also reported by Negash and Bekele (2017) and Nalivata et al. (2017) in Ethiopia and Malawi, respectively.

### Economically Optimal Nutrient Rates for N over a Varying Range of Fertilizer Use Cost to Grain Price Ratios

This study recorded EOR^N^ of 61 kg N ha^−1^, which is 32% less than the recommended rate of 90 kg ha^−1^ N required for maize production in the semi-deciduous forest AEZ of Ghana. This means the farmer can save 29 kg ha^−1^ of the fertilizer cost instead of 90 kg N ha^−1^ since application beyond 61 kg N ha^−1^ is uneconomical. The 61 kg N ha^−1^ EOR^N^ for maize obtained in this study as compared to the higher values reported by Tetteh et al. (2017) could have been due to the rainfall distribution pattern recorded during the experimental period coupled with the significantly (*p* < 0.05) greater N uptake observed after the application of 60 kg N ha^−1^ (Table 5). The semi-deciduous forest zone is generally characterized by having average annual rainfall amounts of 1500 mm as compared to the transitional and savannah zones which have an average annual rainfall of 1300 mm and 1000 mm, respectively. The rainfall received by the crop may have resulted in faster nutrient dissolution and translocation into the various parts of the maize crop, leading to higher grain yields of maize and N uptake.

From the observed results, higher net returns of US$348.77 could be obtained at the EOR^N^ of 61 kg ha^−1^. The results obtained are consistent with that of Jansen et al. (2013) who reported that excessively high fertilizer cost causes uneconomical fertilizer costs to grain price ratios (CP) and low net returns to fertilizer use. The results of the EOR^N^ obtained in this study are more economical than the ones obtained by other researchers in sub-Saharan Africa, where the EOR^N^ was 10–100% greater than the recommended rate of N for maize (Cyamweshi et al. 2017; Kibunja et al. 2017). The EOR^N^ recorded by Tetteh et al. (2017) were approximately 1% (89 kg N ha^−1^) and 23% (69 kg N ha^−1^) less than the 90 kg N ha^−1^ recommended rate for the Forest/Savanna transition and Southern Guinea Savanna zones of Ghana, respectively.

## Conclusion

This study has successfully used the Optimizing Fertilizer Recommendation for Africa (OFRA) tool to predict an economically optimal N rate (EOR^N^) for maize in the semi-deciduous forest zone of Ghana. This implies that, the OFRA fertilizer optimization tool has the potential as a decision support tool, to optimize the best returns on the minimal investments in fertilizer use, by predicting and selecting the best crop-nutrient combination(s) resulting in increased net returns. Furthermore, however, interventions that reduce fertilizer cost to grain price ratios are needed as it enhances the profitability of fertilizer use and increase the capacity of the financially constrained smallholder farmer to invest in fertilizer use.

## Electronic supplementary material

ESM 1 (DOCX 23 kb)
