# Supplementary material for: Economically Optimal Rate for Nutrient Application to Maize in the Semi-deciduous Forest Zone of Ghana
Source: J Soil Sci Plant Nutr. 2020 Apr 17;20(4):1703–13. doi: 10.1007/s42729-020-00240-y (PMC7655581; doi:10.1007/s42729-020-00240-y)
Supplement: Supplementary file 1 — (DOCX 23 kb) [file 42729_2020_240_MOESM1_ESM.docx]

**Economically optimal rate for nutrient application to maize in the semi-deciduous forest zone of Ghana**

Journal of Soil Science and Plant Nutrition

Benedicta Essel^1,2^, Robert Clement Abaidoo^3,4^, Andrews Opoku^1^ and Nana Ewusi-Mensah^1^

Corresponding author: Benedicta Essel (esselbenedicta@yahoo.com)

^1^Department of Crop and Soil Sciences, Faculty of Agriculture, Kwame Nkrumah University of Science and Technology (KNUST), Kumasi, Ghana

^2^CSIR - Soil Research Institute, Academy Post Office, Kwadaso, Kumasi, Ghana

^3^Department of Theoretical and Applied Biology, Kwame Nkrumah University of Science and Technology (KNUST), Kumasi, Ghana

^4^International Institute of Tropical Agriculture, PMB 5320, Oyo Road, Ibadan

Online Resource 1. Rainfall pattern at the experimental site during the major season of 2017

Online Resource 2. Rainfall pattern at the experimental site during the minor season of 2017
